# Biochemical and immunological mechanisms by which sickle cell trait protects against malaria

**DOI:** 10.1186/1475-2875-12-317

**Published:** 2013-09-11

**Authors:** Lauren Gong, Sunil Parikh, Philip J Rosenthal, Bryan Greenhouse

**Affiliations:** 1 University of California Berkeley-University of California San Francisco Joint Medical Program, Berkeley, CA, USA; 2 Yale University, New Haven, CT, USA; 3 University of California, Box 1234, San Francisco 94143, CA, USA

**Keywords:** Malaria, *Plasmodium falciparum*, Sickle cell trait, Genetics, Red blood cell polymorphisms, Sickle haemoglobin, Immunology, Immunopathogenesis, Protection

## Abstract

Sickle cell trait (HbAS) is the best-characterized genetic polymorphism known to protect against falciparum malaria. Although the protective effect of HbAS against malaria is well known, the mechanism(s) of protection remain unclear. A number of biochemical and immune-mediated mechanisms have been proposed, and it is likely that multiple complex mechanisms are responsible for the observed protection. Increased evidence for an immune component of protection as well as novel mechanisms, such as enhanced tolerance to disease mediated by HO-1 and reduced parasitic growth due to translocation of host micro-RNA into the parasite, have recently been described. A better understanding of relevant mechanisms will provide valuable insight into the host-parasite relationship, including the role of the host immune system in protection against malaria.

## Background

Malaria, especially that caused by *Plasmodium falciparum*, has been a major cause of morbidity and mortality throughout human history. As a result, malaria has exerted extraordinary evolutionary pressure on the human genome and appears to have selected for multiple genetic polymorphisms that provide protection against severe disease [1-4]. The best-characterized human genetic polymorphism associated with malaria results in sickle haemoglobin (HbS). The high prevalence of HbS in sub-Saharan Africa and some other tropical areas is almost certainly due to the protection against malaria afforded to heterozygotes [1-3,5]. Since the protective effect of sickle cell trait on malaria was first described over 60 years ago [6-8] our understanding of the epidemiology and mechanisms of protection of this genotype have continued to expand, as will be discussed below.

### Sickle haemoglobin, sickle cell disease and sickle cell trait

Sickle haemoglobin (HbS) is a structural variant of normal adult haemoglobin. Adult haemoglobin (HbAA) is made up of two alpha and two beta globin chains. HbS is the result of a single point mutation (Glu → Val) on the sixth codon of the beta globin gene [9]. Homozygotes for haemoglobin S (HbSS) with two affected beta chains develop sickle cell disease, in which polymerized haemoglobin causes red blood cells to sickle and occlude blood vessels. Vaso-occlusion affects many organs and tissues, and results in high morbidity and mortality. Heterozygotes for sickle haemoglobin (HbAS) have sickle cell trait and are generally asymptomatic [10].

### HbAS and protection against malaria

Despite the obvious deleterious nature of HbSS, it is now widely accepted that the persistence of the sickle mutation in human populations is due to the protection from malaria afforded to heterozygous individuals. Haldane first proposed the concept of a heterozygote advantage against malaria in 1949 [11]. In this seminal paper, Haldane suggested that individuals heterozygous for thalassaemia, another haemoglobinopathy, were protected against malaria.

Contemporaneous to this hypothesis, epidemiologic evidence for protection against malaria in those with HbS was emerging. In 1946, a British medical officer reported that the prevalence of malarial parasitaemia was lower in a cohort of 'sicklers” compared to “non-sicklers” in Northern Rhodesia [6], findings corroborated in 1952 [8]. The first true test of Haldane’s hypothesis came in 1954 through the work of Alison linking HbAS to protection against malaria in Uganda [7]. This study showed a reduced prevalence of parasitaemia (particularly *P. falciparum*) in Ugandans with HbAS compared to those with HbAA. In addition, after experimental infection Ugandans with HbAS had reduced parasitaemia and less clinical malaria than those with HbAA. Since these observations, strong evidence for the protective effects of HbAS against malaria has been generated in multiple case control and cohort studies [12-17]. Recently, an evidence-based map of the global distribution of the sickle cell variant has been created in a bayesian geostatistical framework and compared to the global prevalence of *P. falciparum*[5]. These data provide comprehensive evidence for a global geographical association between malaria burden and HbS allele frequency, particularly in sub-Saharan Africa. Furthermore, evidence for the protective effects of other red blood cell polymorphisms against malaria, including haemoglobin C, haemoglobin E, thalassaemias, and ovalocytosis have also been described [17-28].

In endemic countries, infection with *P. falciparum* causes a range of outcomes, including asymptomatic parasitaemia, uncomplicated disease and severe malaria, which commonly progresses to death. HbAS provides significant protection against both severe and uncomplicated malaria. Case–control and cohort studies in multiple African countries have consistently found that HbAS is 70-90% protective against severe malaria [12-16] and 75% protective against hospitalization for malaria [29]. A recent meta-analysis reviewed 44 studies of children with HbAS and reported significant protection from severe malaria syndromes, including greater than 90% protection from severe malaria, cerebral malaria and severe malarial anaemia [17]. In addition, a cohort study showed a 60% reduction in overall mortality in HbAS children aged two to 16 months, compared to HbAA children, in an area of high malaria transmission [30]. Children with HbAS are also protected from uncomplicated malaria, with cohort studies showing that HbAS is 30-50% protective [15,17,27,31-36].

While associations between HbAS and protection against malaria are clear, data from clinical studies aiming to identify mechanism(s) of protection have been less consistent. Older studies found a lower prevalence of parasitaemia in HbAS individuals irrespective of symptoms [7,37], suggesting HbAS exerts protection against the establishment of parasitaemia. Multiple other reports failed to identify an association between HbAS and the prevalence of asymptomatic parasitaemia [29,31,38-40], but three recent studies found that HbAS children had significantly less asymptomatic parasitaemia than HbAA children [41-43]. Further, HbAS children in Ghana had significantly lower parasite densities and a higher proportion of submicroscopic *P. falciparum* infection compared to HbAA children [41]. Data on associations between HbAS and the multiplicity of infection, the number of genetically distinct parasites causing an infection, are limited and results have been conflicting [26,35,44,45]. A potential reason for these discrepancies is that, depending on the epidemiological context, high multiplicity of infection may reflect either lack of protection against infection, allowing the establishment of a larger number of patent parasites, or protection against symptomatic disease, allowing parasite clones to “stack up” since patients are less likely to seek care and receive antimalarial therapy. To further investigate the effect of HbAS on parasitaemia, another study followed a cohort of Ugandan children aged one to ten years for asymptomatic parasitaemia and symptomatic malaria, using genotyping to detect and follow individual parasite clones longitudinally [35]. This study found that HbAS protected against the establishment of parasitaemia by decreasing the force of infection, or the average number of parasite strains causing blood stream infections, and the probability of developing clinical symptoms once parasitaemic. HbAS children were also protected against high parasite densities during symptomatic malaria, consistent with prior studies [26,29-31,33,35,41,46], likely contributing importantly to protection against severe malaria. These discrepancies suggest that the mechanism of protection afforded by HbAS is complex, with impacts on both the development of parasitaemia and the control of parasitaemia once it is established.

### Molecular mechanism of protection

Some decades ago, investigators found that *P. falciparum* parasites induced sickling of HbAS red blood cells *in vitro*. In the 1970s, two groups showed that parasitized HbAS cells sickled at a two to eight times higher rate than non-parasitized cells [47,48]. One group also visualized polymerized haemoglobin in parasitized red blood cells and hypothesized that an increase in the polymerized haemoglobin or a reduced intracellular pH might cause increased sickling [48]. Increased sickling of parasitized red blood cells in HbAS individuals may promote enhanced phagocytosis of infected cells and, therefore, result in reduced parasitaemia compared to that in HbAA individuals (Table 1).

**Table 1:** Hypothesized protective mechanisms of sickle cell trait (HbAS) against malaria

**Hypothesized mechanism of protection**		**Evidence**	
**Biochemical**	HbAS changes how *P. falciparum* establishes an infection in the human host	*P. falciparum* induces sickling of red blood cells	Luzzatto 1970; Roth 1978
		Reduced intra-erythrocytic growth of *P. falciparum* in HbAS red blood cells	Friedman 1978; Pasvol 1978; Roth 1978; La monte 2012
		Reduced *P. falciparum* invasion of HbAS red blood cells	Luzatto 1970
	*P. falciparum* induces changes in the red blood cell resulting in altered disease progression.	Reduced rosette formation	Carlson 1999
		Reduced cytoadherence	Cholera 2008
**Immunological**	Improved innate immune response	Enhanced phagocytosis of parasitized HbAS red blood cells	Ayi 2004; Urban 2006; Lang 2009
	Improved acquired immune response	Epidemiologic evidence of an increase in protection against malaria with age in HbAS children	Guggenmoss-Holzmann 1981; Williams 2005; Gong 2012
	Increased cell mediated immune response	Increased lymphoproliferative response in HbAS children	Bayoumi 1990; Abu Zeid 1991; Abu Zeid 1992; Le Hesran 1999
	Increased humoral immune response	Higher IgG levels in HbAS individuals	Edozien 1960; Cornille-Brogger 1979; Verra 2008
		Higher levels of antibodies toward PfEMP-1 in HbAS individuals	Marsh 1989; Cabrera 2005; Verra 2008
	Modulation of immunopathogenesis	Increased levels of HO-1 reduce inflammation irrespective of parasite load.	Ferreira 2011

Later in the 1970s, multiple studies found that *P. falciparum* ring-stage parasites did not grow in HbAS red blood cells under low oxygen tension [48-50]. Parasite growth was inhibited in both sickled and non-sickled HbAS red blood cells [50] suggesting that factors in addition to sickling affected parasite growth. It has been hypothesized that specific intra-erythrocytic conditions of HbAS red blood cells, such as low intracellular potassium [49], high concentrations of haemoglobin [51] or osmotic shrinkage of the red blood cell [52] cause an inhospitable environment for parasites. A study also demonstrated that *P. falciparum* parasites invaded HbAS red blood cells less efficiently than HbAA cells at low oxygen tension [47] but subsequent studies failed to replicate these results [53]. Recent data provide support for the intriguing possibility that human micro RNAs translocated into parasite mRNA reduce intra-erythrocytic growth. This study found two human micro RNAs that were highly enriched in erythrocytes with HbAS, and these micro RNAs inhibited translation of specific parasite mRNA transcripts negatively impacting parasite growth *in vitro*[54].

Biochemical and mechanical changes in infected HbAS red blood cells have been shown to alter disease progression. Rosette formation, which is the binding of *P. falciparum*-infected red blood cells to uninfected red blood cells, is thought to lead to microcirculatory obstruction in cerebral malaria [55-59]. Rosette formation was found to be impaired in *P. falciparum*-infected HbAS red blood cells under deoxygenated conditions [57]. Impaired rosette formation with HbAS red blood cells may be due to increased sickling of these cells in deoxygenated conditions [47,48] or to reduced expression of erythrocyte surface adherence proteins [60]. Decreased rosette formation and the resulting decreased circulatory obstruction might contribute to protection against severe malaria in HbAS individuals.

Reduced cytoadherence has also been implicated as a mechanism of protection in HbAS individuals. Infected red blood cells express one of a family of parasite-encoded *P. falciparum* erythrocyte membrane protein 1 (PfEMP-1) molecules on the erythrocyte surface, and via this protein adhere to endothelial cells in the microvasculature [61-64] .This process, termed cytoadherence, enables parasites to sequester in the vasculature and avoid clearance by the spleen [64]. Cytoadherence also leads to endothelial activation and associated inflammation in the brain and other organs, important in the progression to severe malaria [65-68]. Reduced cytoadherence was first seen in infected red blood cells with another haemoglobinopathy, HbC, [69] which is due to a different mutation (Glu → Lys) in the same codon on the beta chain affected in HbS. Altered expression of PfEMP-1 was subsequently found in HbAS red blood cells *in vitro*[60]. Comparison of binding properties showed reduced adherence to endothelial cells expressing the binding ligand CD36 compared to HbAA red blood cells. PfEMP-1 surface signal was reduced by 14 % in HbAS and HbSS compared to HbAA erythrocytes in flow cytometric assays, suggesting altered surface expression of PfEMP-1, similar to that reported in HbC [60]. In addition, dysfunctional cytoskeletons have been visualized in HbSC erythrocytes [70]. Oxidized haemoglobin present in erythrocytes containing sickled haemoglobin may interfere with actin re-organization in infected HbSC erythrocytes leading to impaired vesicular transport of PfEMP-1 to the erythrocyte surface membrane [70]. These changes may impair parasite-induced remodelling of the red blood cell surface membrane and lead to altered PfEMP-1 surface expression [60,69]. Reduced cytoadherence of HbAS and HbSS erythrocytes likely leads to increased splenic clearance, and may in part explain lower parasite densities and a lower incidence of severe malaria in HbAS individuals.

### Role of the innate immune system

Phagocytosis by monocytes of HbAS red blood cells infected with ring-stage *P. falciparum* was found to be enhanced compared to that of infected HbAA cells, providing evidence for a role of the innate immune system in protection against *P. falciparum* in HbAS individuals [53]. Enhanced phagocytosis may be due to increased presentation of opsonins, including membrane bound IgG, C3c, membrane-bound hemichromes, and aggregated band 3 [53]. These opsonins, which are thought to be involved in the removal of senescent red blood cells, were first shown to be increased in G6PD deficiency [71,72], a red blood cell enzyme deficiency also protective against malaria [1] and were also significantly higher in infected HbAS compared to HbAA red blood cells. Clearance by monocytes of red blood cells with exposed phosphatidylserine, a surface marker of damaged erythrocytes [73-77], was also enhanced in infected HbAS compared to HbAA cells [73].

Enhanced opsonization and clearance of parasitized HbAS red blood cells by the spleen may lead to increased antigen presentation and earlier development of acquired immunity compared to that in HbAA individuals. A cross-sectional study found decreased levels of peripheral myeloid dendritic cells and monocytes in individuals with HbAS during healthy periods and malaria [78], suggesting increased monocyte and dendritic cell recruitment to the spleen.

### Role of the acquired immune system

Population studies have found that the protective effect of HbAS increases with age, suggesting an acquired component of protection. A cross-sectional study of children with malaria in Nigeria found a significantly lower mean parasite density in HbAS compared with HbAA children in those two to four years old, but not in children less than two years old [476]. In Kenyan children, protection afforded by HbAS against symptomatic malaria increased from 20 % in children less than two years old to a peak of 56 % by age ten years [15]. In a recent study, protection against the establishment of parasitaemia and the development of symptomatic malaria once parasitaemic significantly increased between the ages of two and nine years [35]. Several studies shed light on possible immune bases for acquired protection.

### Role of cell mediated immunity

Cell mediated responses to *P. falciparum* appear to be increased in HbAS compared to HbAA individuals. The mean lymphoproliferative response to affinity-purified *P. falciparum* soluble antigens [79-81] was found to be significantly higher in HbAS children compared to HbAA children [82-85] but a significant difference has not consistently been found between HbAA and HbAS adults [82,83]. Thus, available results suggest a more robust cellular response to *P. falciparum* in HbAS children. However, it is unclear whether this is a cause or effect of the protective effects of HbAS. The lymphoproliferative response is suppressed during and after acute malarial infection in HbAA individuals [64,81,86-89]. Therefore, a more robust lymphoproliferative response in HbAS individuals could be secondary to protection against malaria from other mechanisms.

### Role of humoral immunity

Investigators have also found evidence for an enhanced humoral response in subjects with HbAS. Increased levels of gamma globulin were found in HbAS compared to HbAA children [90,91]. However, higher levels of specific antibodies directed at parasite surface antigens believed to play a role in protective responses, including Pf155/ring infected erythrocyte surface antigen (RESA), merozoite surface protein 1 (MSP1), merozoite surface protein 2 (MSP2), erythrocyte binding antigen 175 (EBA175), and glutamate-rich protein (GLURP), have not been seen in HbAS compared to HbAA individuals in most studies [31,85,92-96]. Recently, a study using a protein microarray representing 491 *P. falciparum* proteins found no increase in the magnitude or breadth of the *P. falciparum*-specific IgG response [97]. One study did find increased levels of antibodies toward free parasite antigens apical membrane antigen 1 (AMA1), EBA175, MSP1, MSP2, MSP3, circumsporozoite protein (CSP), and parasite schizont extract (PSE) in HbAS *vs* HbAA children living in areas of low malarial transmission in Burkina Faso [98], but not in children in an area of higher transmission. Another study found lower levels of IgG1 and IgG3 to MSP2 and RESA in HbAS individuals [99], possibly due to reduced exposure to these antigens in HbAS individuals.

In contrast, higher levels of IgG directed at PfEMP-1 family proteins, which are located on the surface of the red blood cell, have been found in individuals with HbAS in a number of studies. On study found HbAS individuals had a higher IgG response to the infected red blood cell *in vitro*[100]. In The Gambia [31], Gabon [101], and a low transmission area of Burkina Faso [98], HbAS children had higher levels of IgG antibodies toward PfEMP-1 than did those with HbAA [31]. However, studies in areas of high malaria transmission failed to find increased antibody response to PfEMP-1 in HbAS children [97,98,102] perhaps because in these high malaria transmission areas robust responses were seen in the majority of children. Other possibilities for discrepancies between these studies include methods of antigen preparation and sample size limitations. The identification of high levels of IgG toward PfEMP-1 and not toward other parasite antigens suggests that the enhanced humoral immune response in HbAS individuals may be directed at proteins on the surface of the infected red blood cell. This phenomenon may be due to increased splenic uptake of infected red blood cells in HbAS individuals and therefore improved presentation of surface antigens. In addition, protection against high parasite densities seen in younger ages [26,29,30,35,41,46] may improve the development of acquired immunity, as parasitaemia may interfere with development of effective immune memory [103]. Higher levels of antibodies to PfEMP-1 may mediate protection in HbAS individuals via enhanced opsonization and phagocytosis of infected red blood cells, or through destabilization of cytoadherence. Accelerated acquisition of antibodies to PfEMP-1 may therefore underly the age-dependent increase in protective effects of HbAS found in three studies [15,35,46]. Alternatively, or in conjunction, antibodies to PfEMP-1 may be more effective in HbAS than in HbAA children due to reduced or altered surface expression of PfEMP-1 levels in the former.

### Modulation of immunopathogenesis

**Figure 1:**
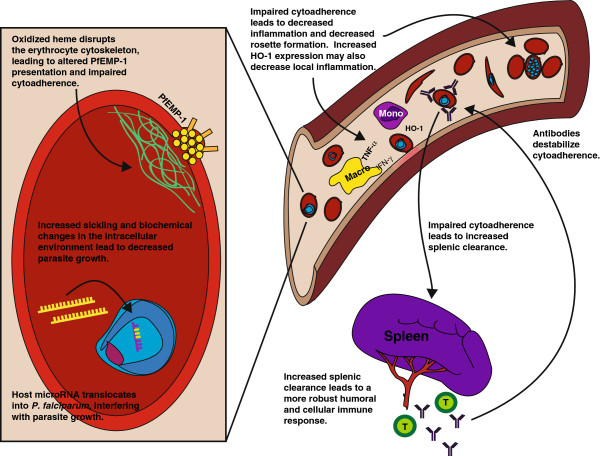
Model of mechanisms by which sickle cell trait may protect against malaria.

In addition to direct antiparasitic effects, HbAS may confer protection against severe malaria by limiting pathogenesis independent of effects on pathogen load. A recent study using a well-established mouse model of sickle cell disease implicated increased levels of hemeoxygenase-1 (HO-1), an enzyme that breaks down free haem, in the mechanism of protection against cerebral malaria [104]. The authors concluded that sickle haemoglobin suppresses the pathogenesis of cerebral malaria by inducing the expression of HO-1 and preventing the accumulation of cytotoxic-free haem, limiting subsequent tissue damage after *Plasmodium berghei* infection. They also reported decreased expansion of cytotoxic CD8 T cells that they hypothesized may also contribute to protection against severe disease. Protection was shown to be present irrespective of parasite density. Although the majority of human studies have shown that HbAS is, in fact, associated with lower parasite densities during symptomatic malaria [26,29-31,33,46] data from this mouse model suggest that modulation of pathogenic host inflammatory responses may be an additional mechanism of protection against severe disease. Figure 1 summarizes a number of these potential mechanisms.

## Conclusions

It is likely that both biochemical and immune mechanisms contribute to the protection afforded against falciparum malaria by the HbAS genotype. HbS clearly induces biochemical changes in the red blood cell that may affect parasite metabolism and growth. *Plasmodium falciparum* also likely inflicts oxidative damage on the HbAS red blood cell. Chronic low levels of oxidized haem in sickled red blood cells may induce HO-1, leading to host tolerance in severe disease. Oxidized haem may also interfere with the formation of an actin cytoskeleton, leading to altered PfEMP-1 expression on the infected red blood cell surface membrane and reduced cytoadherence. Reduced cytoadherence may lead to decreased endothelial activation and decreased inflammation implicated in the pathogenesis of severe malaria. In addition, it could also lead to increased splenic uptake of infected erythrocytes. Within the spleen, increased antigen presentation may lead to accelerated development of protective immune responses, including antibodies to PfEMP-1, potentially augmented by biochemical protection against high parasite densities that might otherwise dampen an effective response. Higher levels of antibodies directed against PfEMP-1 and possibly other surface proteins may enhance opsonization and phagocytosis of infected red blood cells and further destabilize the cytoadherence properties of infected red blood cells. While there are some data to support all of these mechanisms, it is still unclear which ones are relevant *in vivo*. Together, however, it is clear that relevant mechanisms lead to better control of parasitaemia in HbAS children and protect against both uncomplicated and severe malaria. Improved understanding of the mechanisms of protection derived from this single point mutation will give further insight into the host-parasite relationship including how *P. falciparum* interacts with the human immune system.

## Abbreviations

AMA1: Apical membrane antigen 1 (AMA1); CSP: Circumsporozoite protein; EBA175: Erythrocyte binding antigen 175; GLURP: Glutamate-rich protein; HbAA: Normal adult haemoglobin; HbAS: Sickle cell trait; HbC: Haemoglobin C; HbS: Sickle haemoglobin; HO-1: Hemeoxygenase-1; MSP1: Merozoite surface protein 1; MSP1: Merozoite surface protein 2; PfEMP-1: *Plasmodium falciparum* erythrocyte membrane protein 1; PSE: Parasite schizont extract; RESA: Pf155/ring infected erythrocyte surface antigen.

## Competing interests

The authors declare that they have no competing interests.

## Authors’ contributions

LG performed a comprehensive review of the primary literature and drafted the manuscript. SP, PJR and BG reviewed and identified additional primary literature, directed the organization of the manuscript and edited the manuscript. All authors read and approved the final version of the manuscript.
